# A case report of acute acalculous cholecystitis due to *Salmonella Paratyphi B* complicated by biliary peritonitis

**DOI:** 10.11604/pamj.2013.16.127.1856

**Published:** 2013-11-30

**Authors:** El Bachir Benjelloun, Leila Chbani, Iman Toughrai, Abdelmalek Ousadden, Khalid Mazaz, Kahlid Ait Taleb

**Affiliations:** 1 Department of surgery, University hospital Hassan II, Morocco; 2 Department of Radiology, University hospital Hassan II, Morocco

**Keywords:** Acalculous cholecystitis, Salmonella Paratyphi B, biliary peritonitis, ultrasonography

## Abstract

Non-typhoidal salmonella are a rare case of acute acalculouscholecystitis (AAC). *Salmonella Paratyphi B*, which accounts for one of the less invasive NTS serotypes, has rarely been reported to cause cholecystitis. We describe a case of 65-year old previously healthy man, who present with signs of acute abdomen, due to biliary peritonitis as a complication of acute acalculouscholecystitis caused by *Salmonella paratyphi B*. Our case illustrates the potential severity of infection with *Salmonella Paratyphi B* especially in older patient. High index of awarenessshould be considered in endemic areas.

## Introduction

Acute acalculouscholecystitis (AAC) is an acute inflammation of the gall bladder in the absence of gallstones. AAC comprises 2-15% of all cases of acute cholecystitis, and it is traditionally known to occur in critically ill patient [1]. AAC occurs in 2% of Salmonella typhi infections. Nontyphoidal salmonella (NTS) are rarely isolated from cases of acute cholecystitis [2]. AAC is believed to have a more fulminate course, frequently associated with gangrene, perforation, and abscess, as well as significantly higher morbidity and mortality especially in older patient. Rapid and accurate diagnosis is essential because ischemia may progress rapidly to gangrene and perforation. If an operation was performed within 48 hours from the onset of symptoms, severe complications would be reduced [3]. We report a case of 65-old- year men, who presented with signs of acute abdomen, due to biliary peritonitis as a complication of an acute acalculouscholecystitis caused by *Salmonella paratyphi B*. This is the rarest case that has been reported, particularly in older patient.

## Patient and observation

A 65-year old previously healthy man, presented to the Emergency Department, with complaints of abdominal pain, nausea and vomiting of 48 hours duration. 7 days later she developed epigastric pain, diarrhoea and high grade fever, she consulting a practitioner who prescribing antibiotic (Thiobactin) and antidiarrheic (Imodium), 2 days before admission, the pain became increasing in nature with nausea and vomiting, so the patient decided to consult in the emergency room at the University Hospital of Hassan II. The patient was not using any specific medication and his medical history did not suggest a major disease. He had no prior history of abdominal surgery or trauma. The patient didn't smoke or drink alcohol.

Physical examination revealed conscious acutely ill man. There was no jaundice. Skin turgor was poor and he appeared dehydrated. The temperature was of 37°C, a pulse rate 90 beat per minute (bpm), a blood pressure 120/70 mm Hg. Abdominal examination revealed mild abdominal distension, general abdominal guarding and rigidity in the right upper quadrant. There were no palpable masses or liver enlargement. The hernial sites were free. On rectal examination the prostate was found to be moderately enlarged.

**Figure 1:**
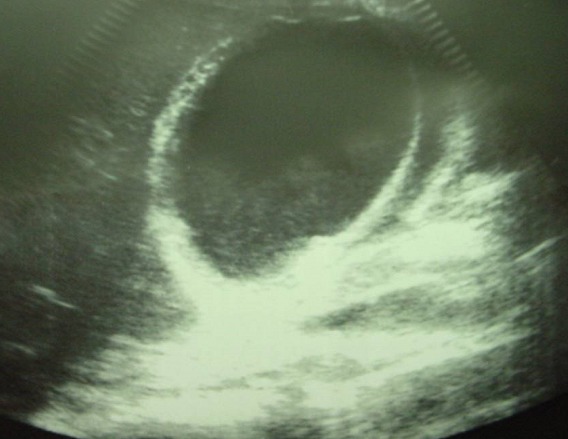
Ultrasonographic image of the gallbladder. Note the distension of the gallbladder, thickening of the wall, pericholecystic fluid collection, and sludge

**Figure 2:**
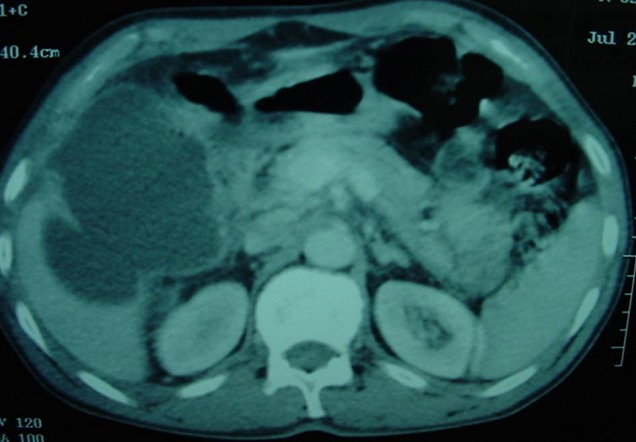
Gallbladder perforation due to acalculouscholecystitis seen at CT

Laboratory data revealed an hematocrit of 41%, an hemoglobin of 12,6g/dl, a white blood cells of 11100/mm^3^ (83% neutrophils), a blood urea of 16 mg/dL, a creatinine level of 0,45 mg/dL. Ultrasonography (US) of the abdomen showed free fluid in the peritoneal cavity, thickening of the gallbladder wall in the absence of gallstones with a pericholecystic collection,.ultrasonographical Murphy′s sign was positive (Figure 1). Computed tomography(CT), confirmed a diagnosis of AAC complicated perforation of the gallbladder and biliary peritonitis, with thickened wall of the gallbladde, pericholecystic collection, and free fluid in the peritoneal cavity (Figure 2). The patient underwent an emergency open cholecystectomy. At exploration, the peritoneal cavity was filled with biliary fluid. The gallbladder was found to be necrotic and perforated with surrounding pus. The pathology of the gallbladder was consistent with gangrenous cholecystitis. Although intra-operative bile cultures did not grow any pathogens, the blood cultures grew *Salmonella Paratyphi B*. Three days after surgery and intravenous antibiotics, the patient general condition improved markedly, he was continued on oral antibiotics and was discharged after 6 days of operation.

## Discussion

Salmonella infections are an important health problem in many countries, mainly those with poor sanitary conditions. Salmonellosis can occur in several different forms: gastroenteritis (the most common syndrome), enteric fever (typhoid fever and paratyphoid fever), bacteremia, chronic carrier state, and localized infections. Localized Salmonella infections are, the most frequently, the result of bacteremia. *Salmonella Paratyphi B* causes enteric fever in humans; the disease closely resembles Typhoid Fever, which is caused by the related serovar *Salmonella Typhi*. *Salmonella Paratyphi B* multiplies in the gastrointestinal tract of humans, and then it penetrates the intestinal mucosa and is transferred via the lymph and the blood to deeper tissues such as the liver and spleen where it survives in macrophages. However, this may result in a generalized or more invasive infection, with secondary septicemia in approximately 3-14% [4].

Acute cholecystitis remains however extremely rare, with a maximal rate of isolation of 0,6% [5]. Ischaemia of the gallbladder and obliteration of the cystic artery are thought to have an important role in the development of AAC. Predisposing factors include age of the patient underlying chronic diseases lymphoproliferatives disorders or diabetes mellitus, organ transplantation and immunosuppressive therapy.

Rapid and accurate diagnosis is essential because ischemia may progress rapidly to gangrene and perforation as we describe in our case. AAC is a surgical emergency and without immediate treatment there may be rapid progression to perforation or gangrenous cholecystitis with mortality as high as 65%. With early diagnosis and intervention, the mortality drops to 7% [6]. Ultrasonography and computed tomography (CT) are the diagnostic imaging procedures of choice for patients with acute AC. Ultrasonography has several advantages. It is portable, noninvasive, and relatively inexpensive. The most significant ultrasonographic criteria for diagnosis include gall bladder thickness of > 3 mm, ultrasonographical Murphy′s sign, enlarged tense gall bladder, pericholecystic fluid, and absence of gall stones [7]; all this criterias were present in our patient.

CT is more sensitive and specific than ultrasound for the diagnosis of AAC, gallbladder perforation and other complications of AAC and its use is appropriate if the gallbladder is inadequately visualised or non-visualised on ultrasound. Cholescintigraphy may demonstrate absence of gall bladder filling in AAC [8].

Emergency cholecystectomy, either open or laparoscopic, is the treatment of choice once the diagnosis of AAC is made, because of the rapid progression of the disease. This is because gangrene and perforation are more frequent and the outcome is worse when compared to acute calculouscholecystitis. But in cases of AAC due to Salmonella infection, conservative management with antibiotics is recommended [9]. For our patient the emergency laparotomy was indicated because AAC was complicated biliary peritonitis. Antibiotics were initiated immediately, when the diagnosis was established.

## Conclusion

In conclusion our case illustrates the potential severity of infection with *Salmonella Paratyphi B* especially in older patient. Early diagnosis, early appropriate antibiotherapy and emergency cholecystectomy are the key to managing AAC due to Salmonella infection.
